# Supplementary figures and images for: Coil-to-α-helix transition at the Nup358-BicD2 interface activates BicD2 for dynein recruitment
Source: eLife. 2022 Mar 1;11:e74714. doi: 10.7554/eLife.74714 (PMC8956292; doi:10.7554/eLife.74714)

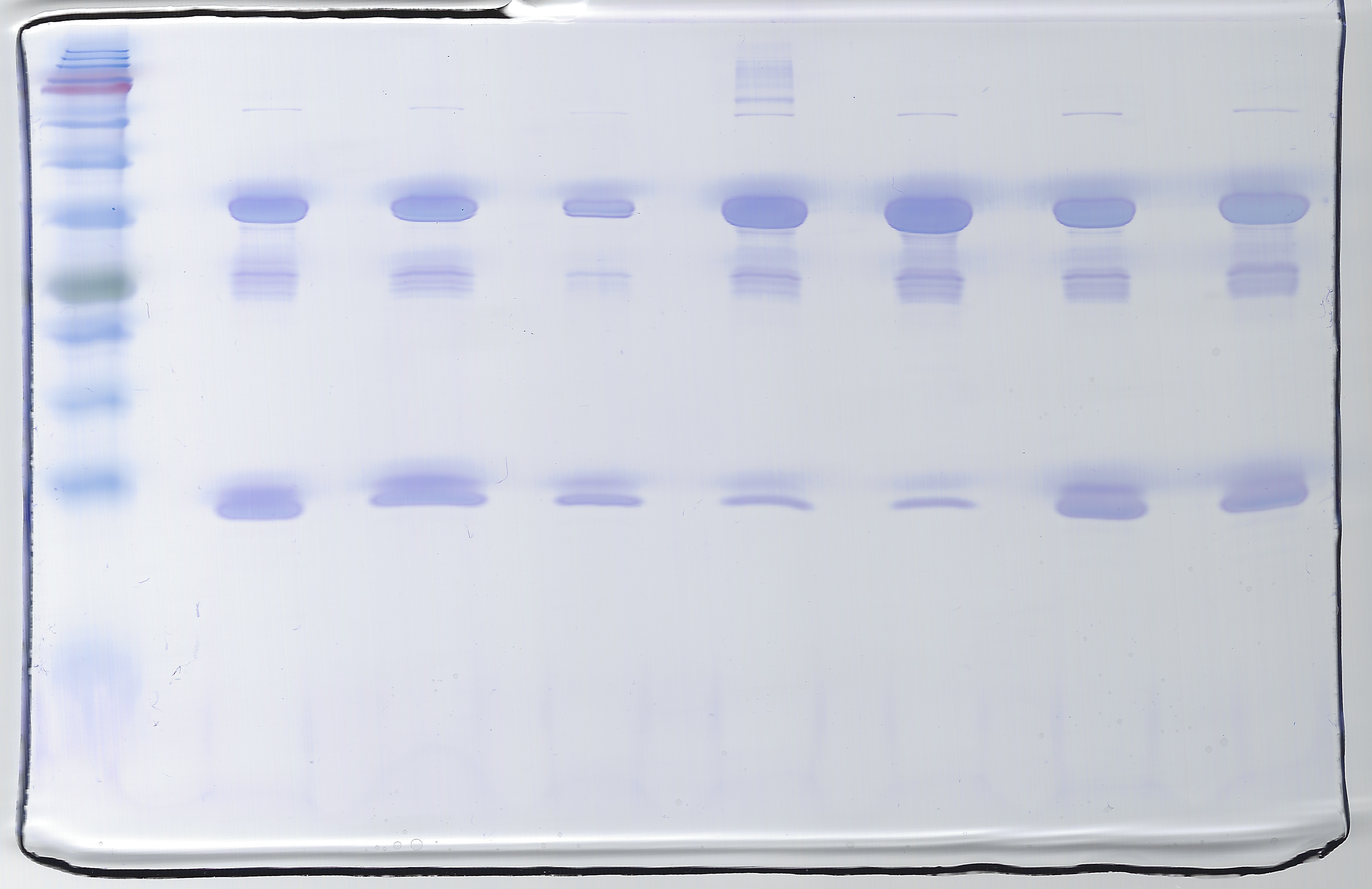

Supplement: Figure 7—source data 2. [file elife-74714-fig7-data2.png]

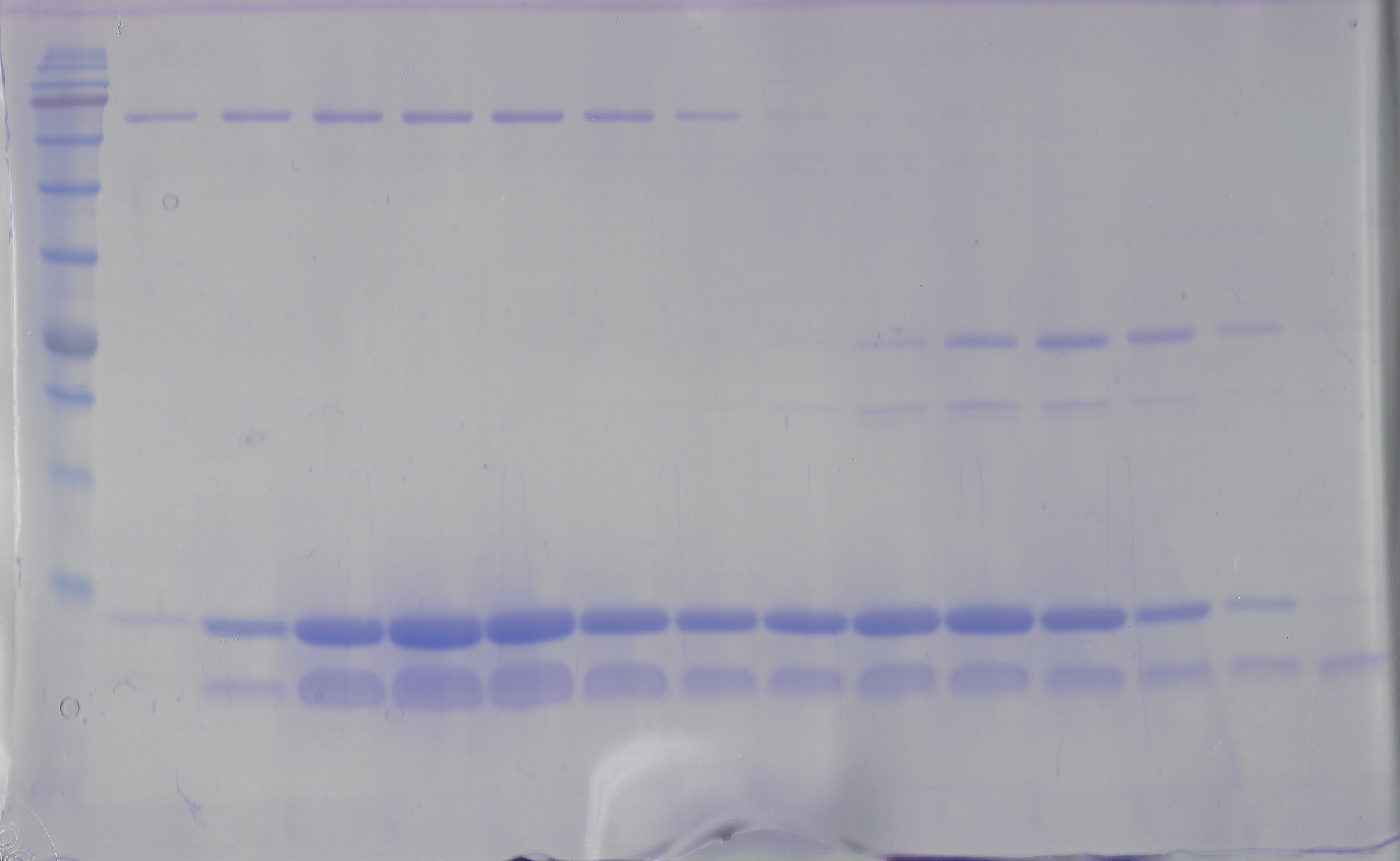

Supplement: Figure 7—source data 3. [file elife-74714-fig7-data3.zip › Figure 7-Source Data 3/Figure 7 - figure supplement 1 C.jpeg]

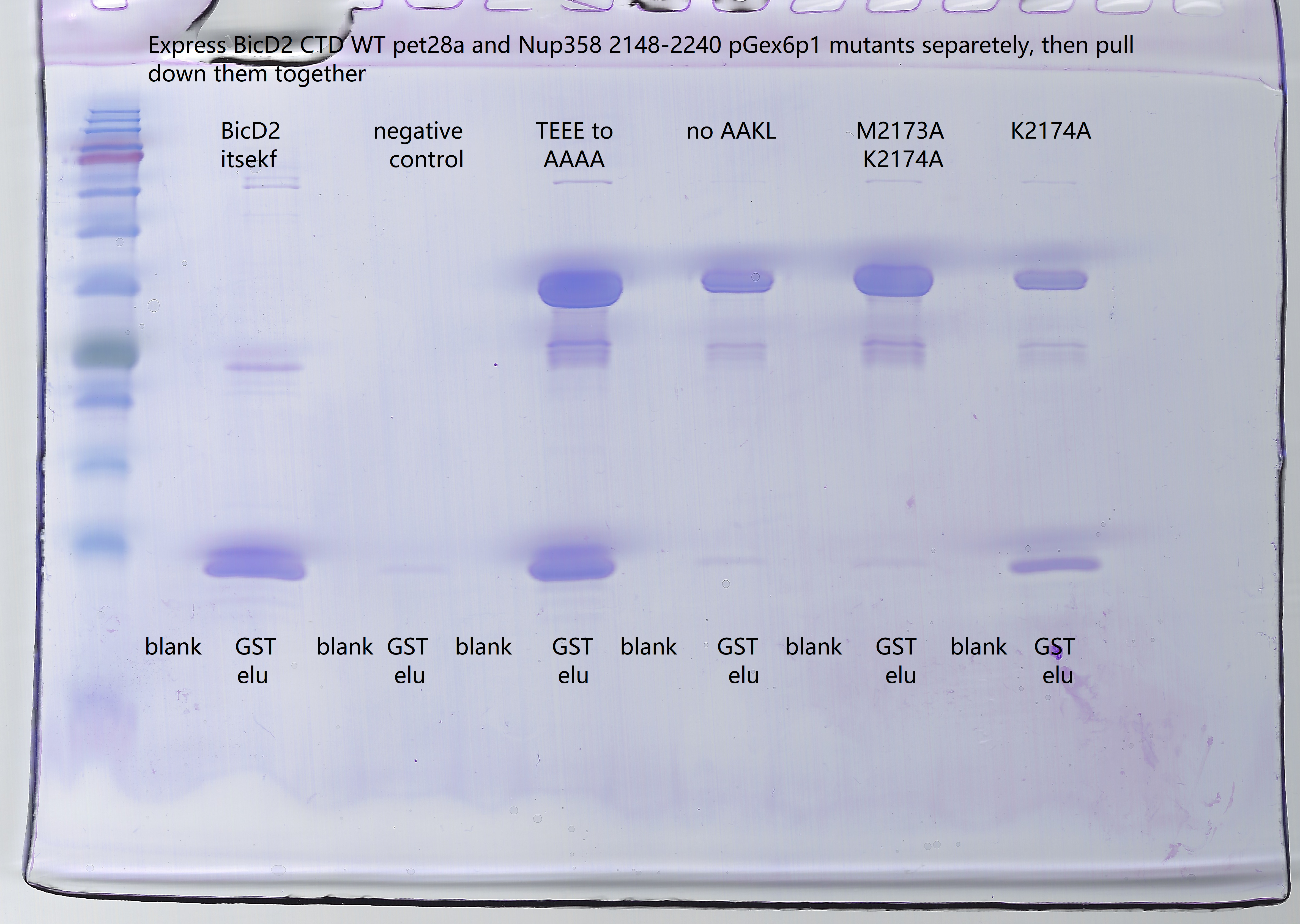

Supplement: Figure 7—source data 3. [file elife-74714-fig7-data3.zip › Figure 7-Source Data 3/Figure 7 - figure supplement 1 B/Figure 7 - figure supplement 1 B_rawdata labelled.png]

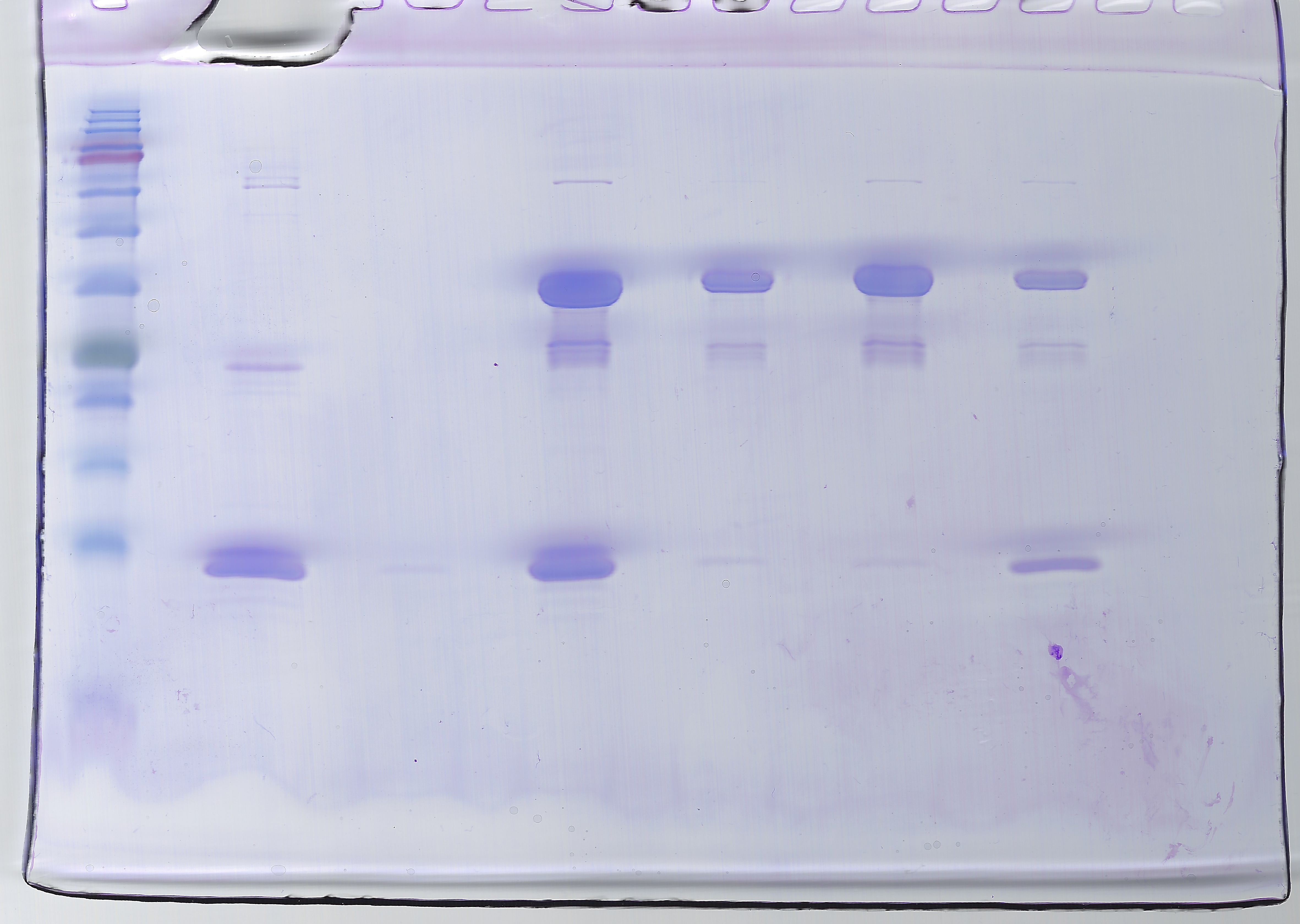

Supplement: Figure 7—source data 3. [file elife-74714-fig7-data3.zip › Figure 7-Source Data 3/Figure 7 - figure supplement 1 B/Figure 7 - figure supplement 1 B.png]

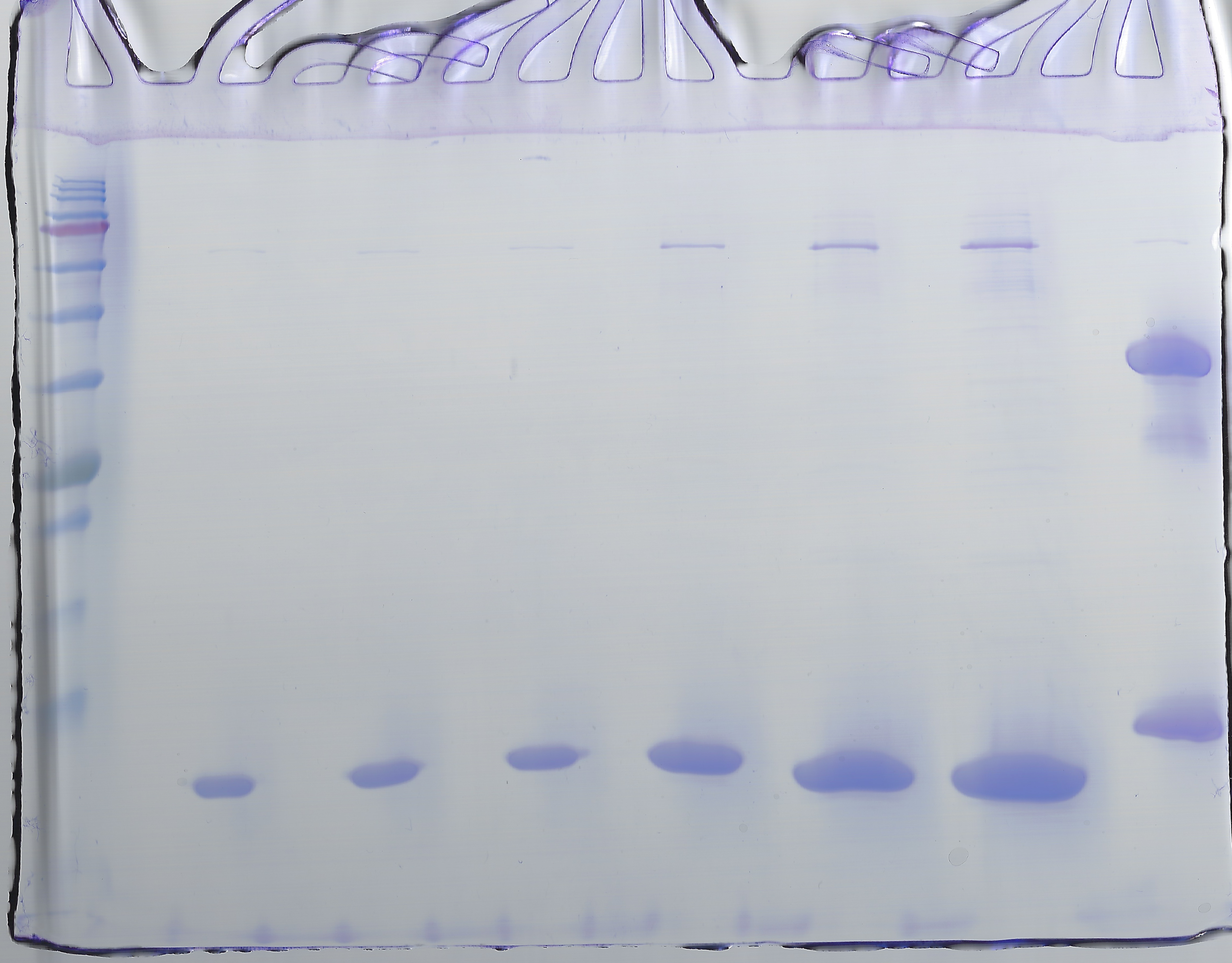

Supplement: Figure 7—source data 3. [file elife-74714-fig7-data3.zip › Figure 7-Source Data 3/Figure 7 - figure supplement 1 D/Figure 7 - figure supplement 1D_rawdata.png]

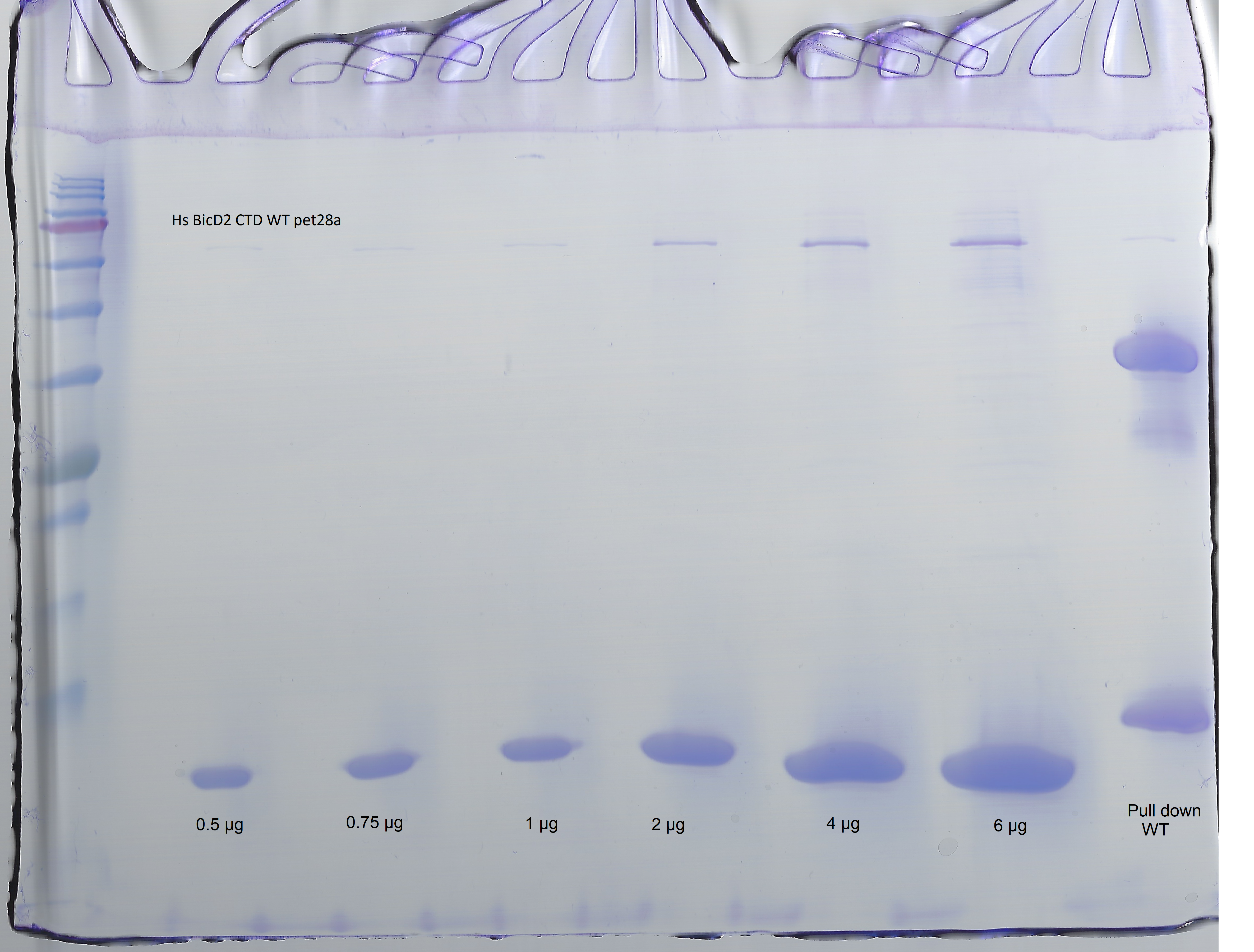

Supplement: Figure 7—source data 3. [file elife-74714-fig7-data3.zip › Figure 7-Source Data 3/Figure 7 - figure supplement 1 D/Figure 7 - figure supplement 1D_rawdata labelled.png]

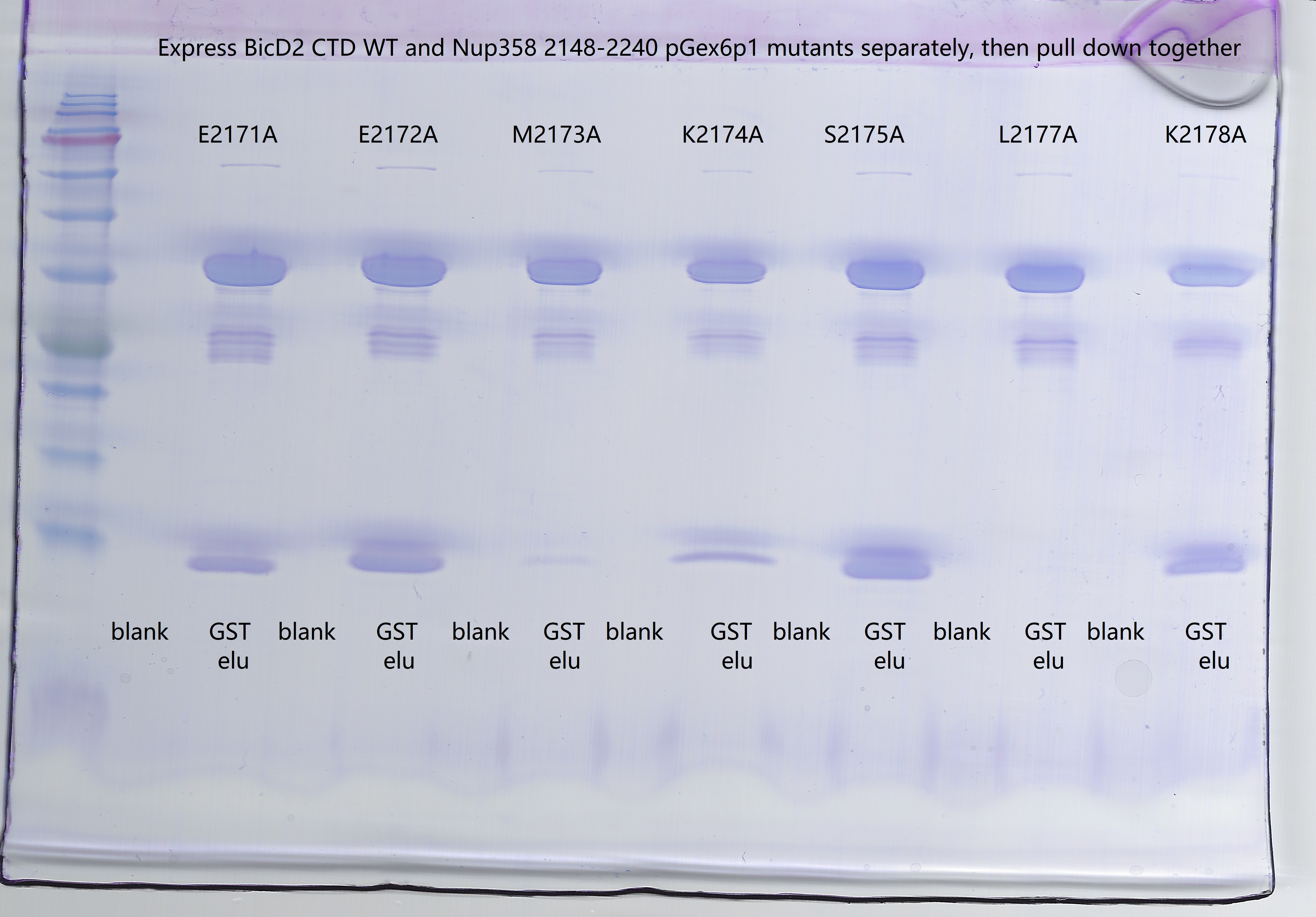

Supplement: Figure 7—source data 3. [file elife-74714-fig7-data3.zip › Figure 7-Source Data 3/Figure 7 - figure supplement 1 A/Figures for replicates/Figure 7 - figure supplement 1 A_rawdata1.2 labelled.png]

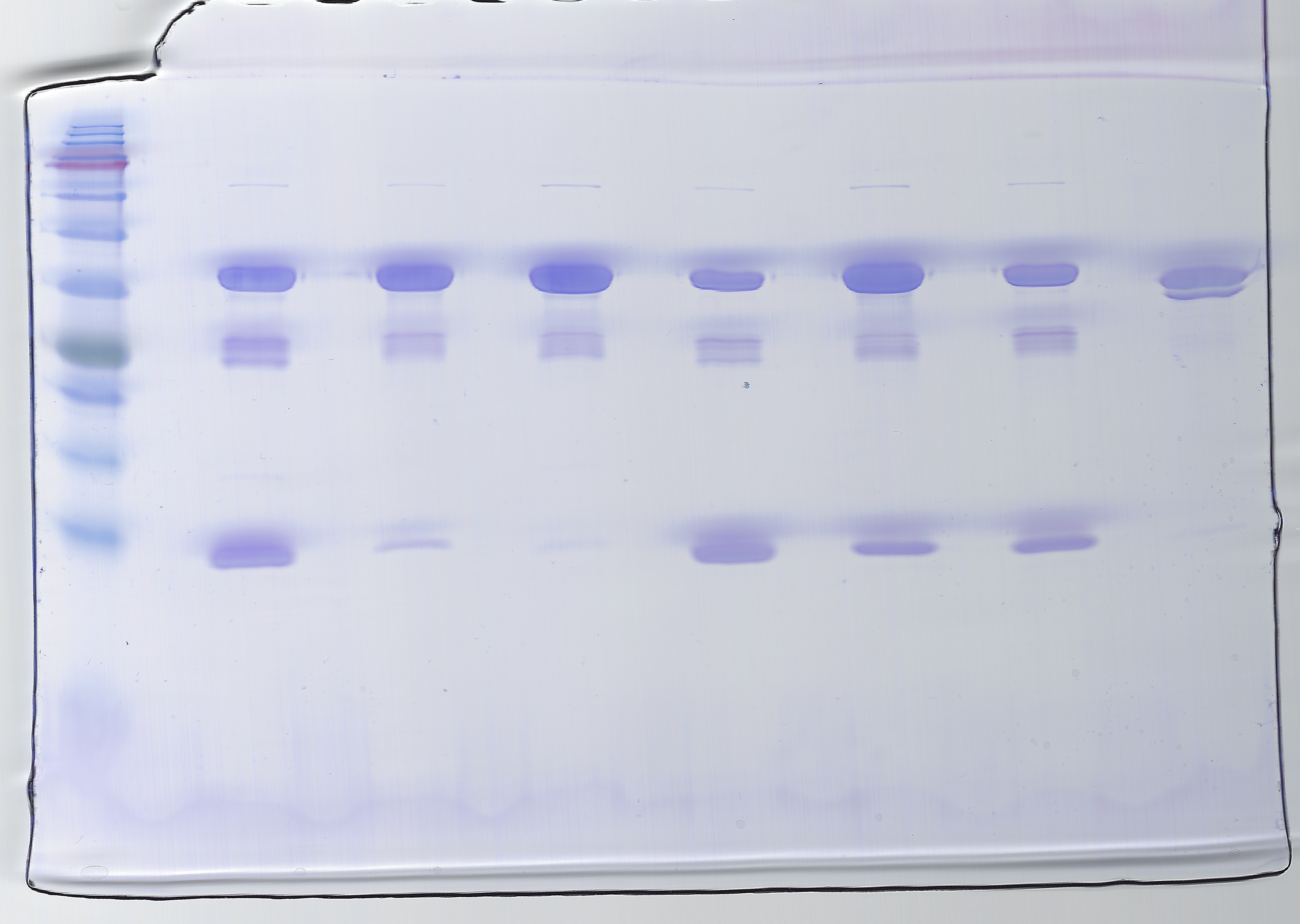

Supplement: Figure 7—source data 3. [file elife-74714-fig7-data3.zip › Figure 7-Source Data 3/Figure 7 - figure supplement 1 A/Figures for replicates/Figure 7 - figure supplement 1 A_rawdata1.3.png]

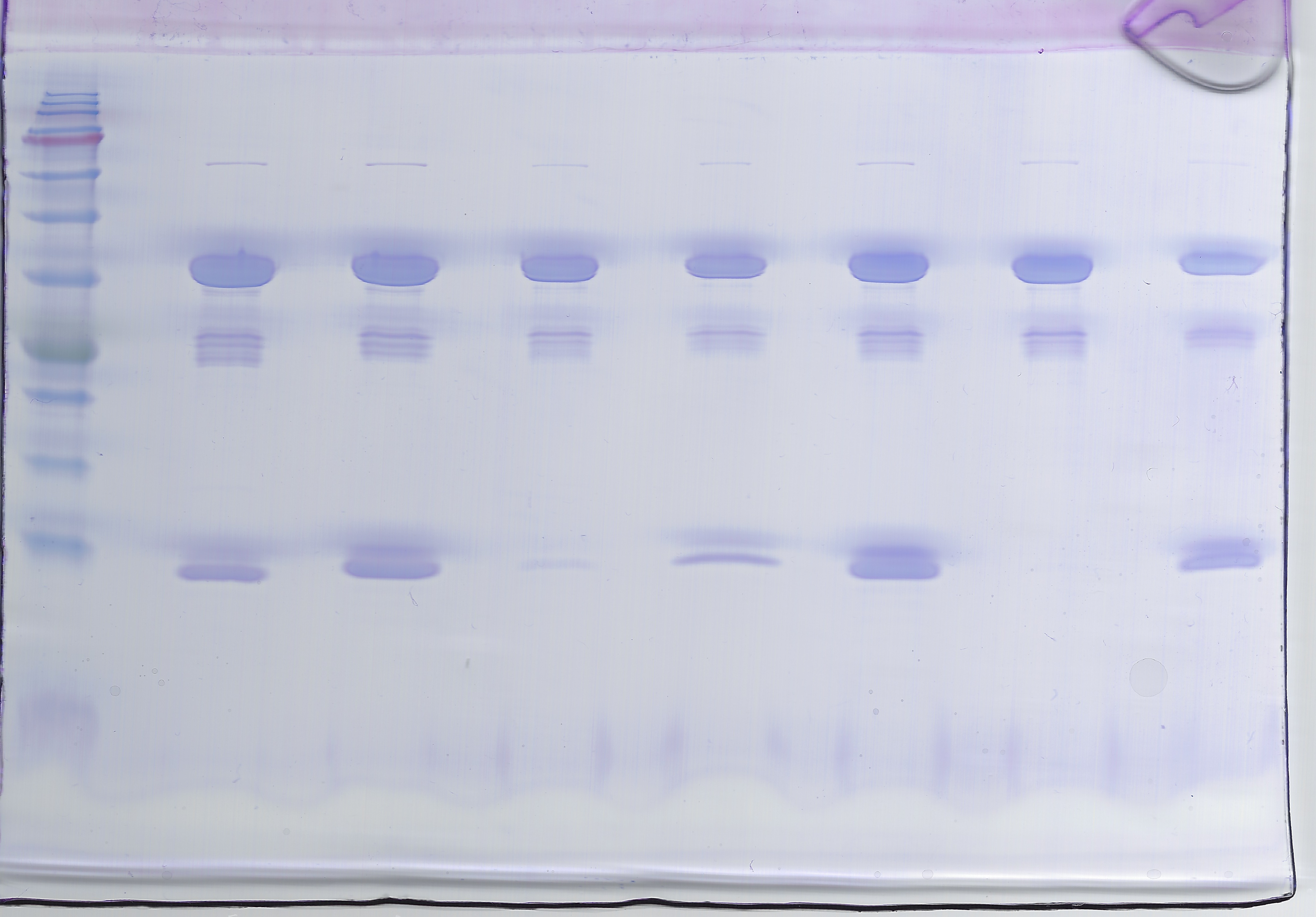

Supplement: Figure 7—source data 3. [file elife-74714-fig7-data3.zip › Figure 7-Source Data 3/Figure 7 - figure supplement 1 A/Figures for replicates/Figure 7 - figure supplement 1 A_rawdata1.2.png]

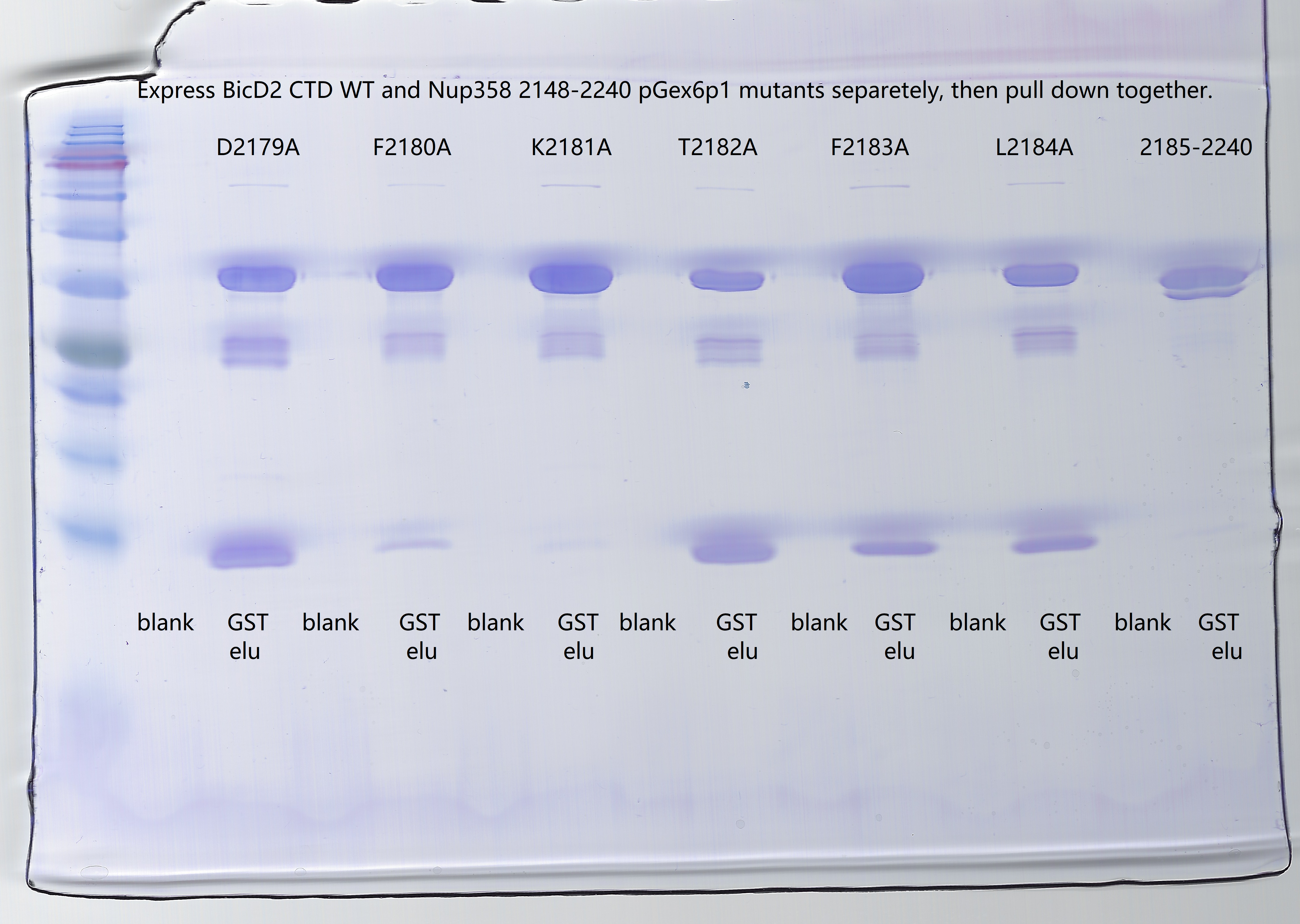

Supplement: Figure 7—source data 3. [file elife-74714-fig7-data3.zip › Figure 7-Source Data 3/Figure 7 - figure supplement 1 A/Figures for replicates/Figure 7 - figure supplement 1 A_rawdata1.3 labelled.png]

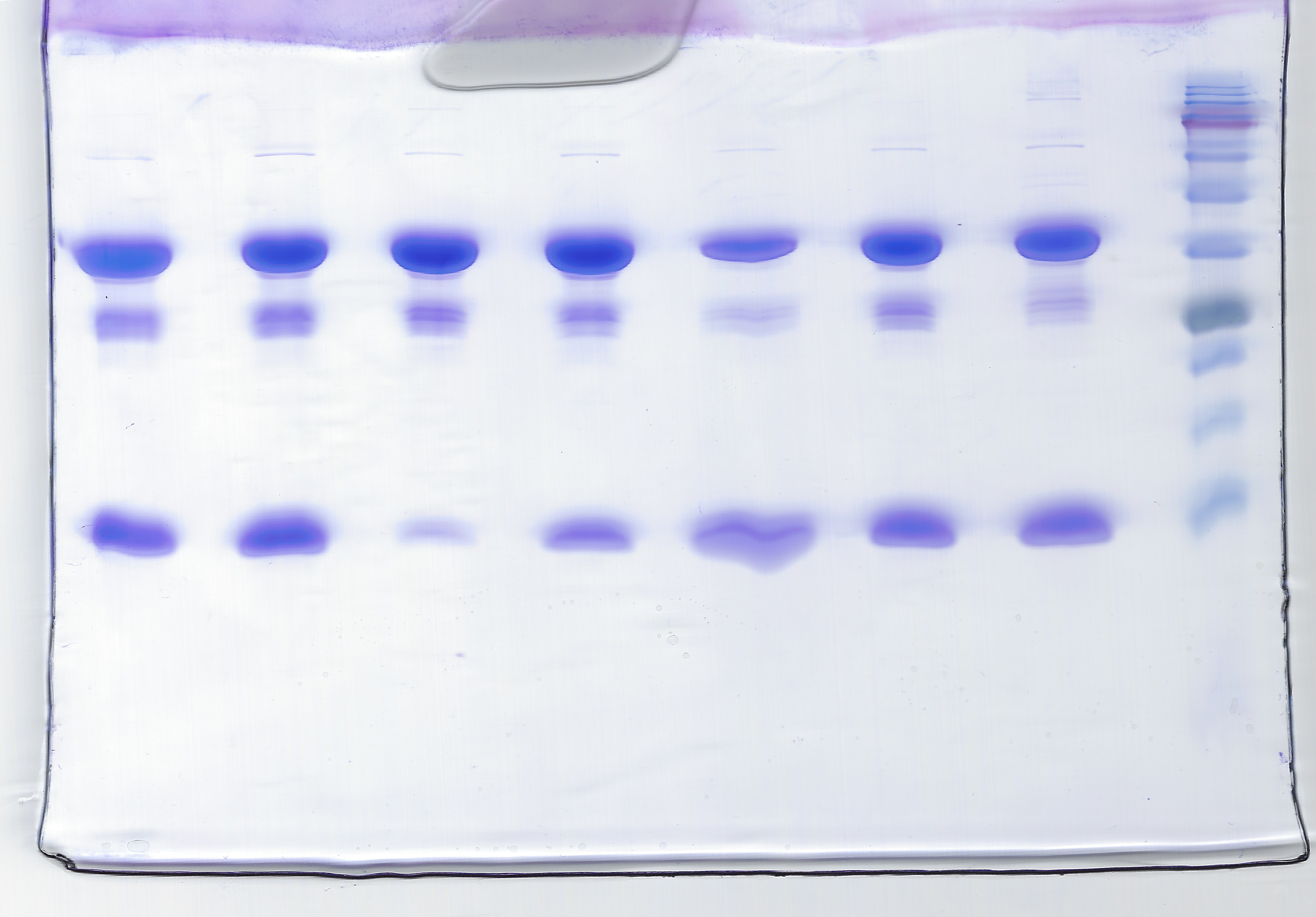

Supplement: Figure 7—source data 3. [file elife-74714-fig7-data3.zip › Figure 7-Source Data 3/Figure 7 - figure supplement 1 A/Figures for replicates/Figure 7 - figure supplement 1 A_rawdata2.1.png]

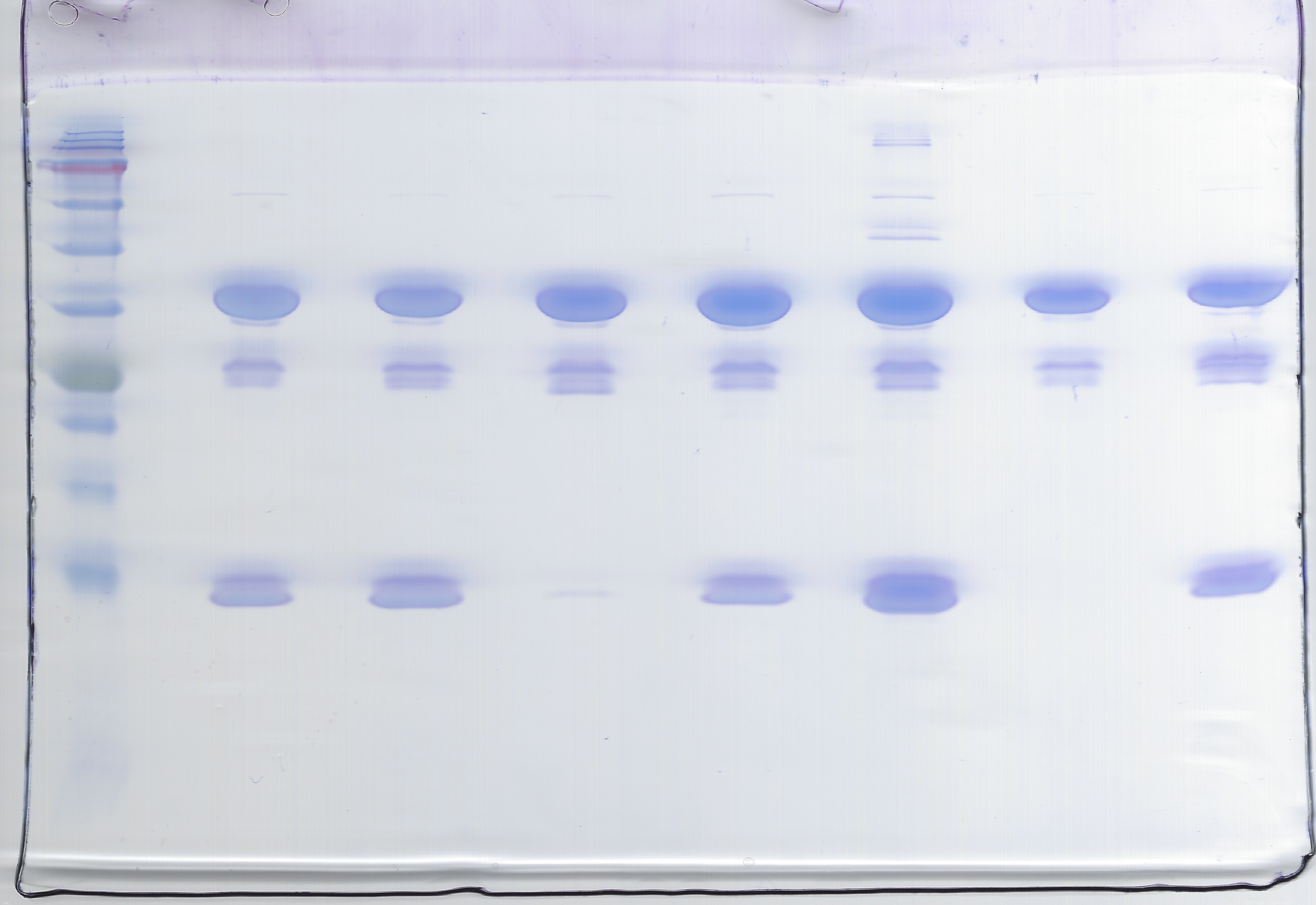

Supplement: Figure 7—source data 3. [file elife-74714-fig7-data3.zip › Figure 7-Source Data 3/Figure 7 - figure supplement 1 A/Figures for replicates/Figure 7 - figure supplement 1 A_rawdata2.2.png]

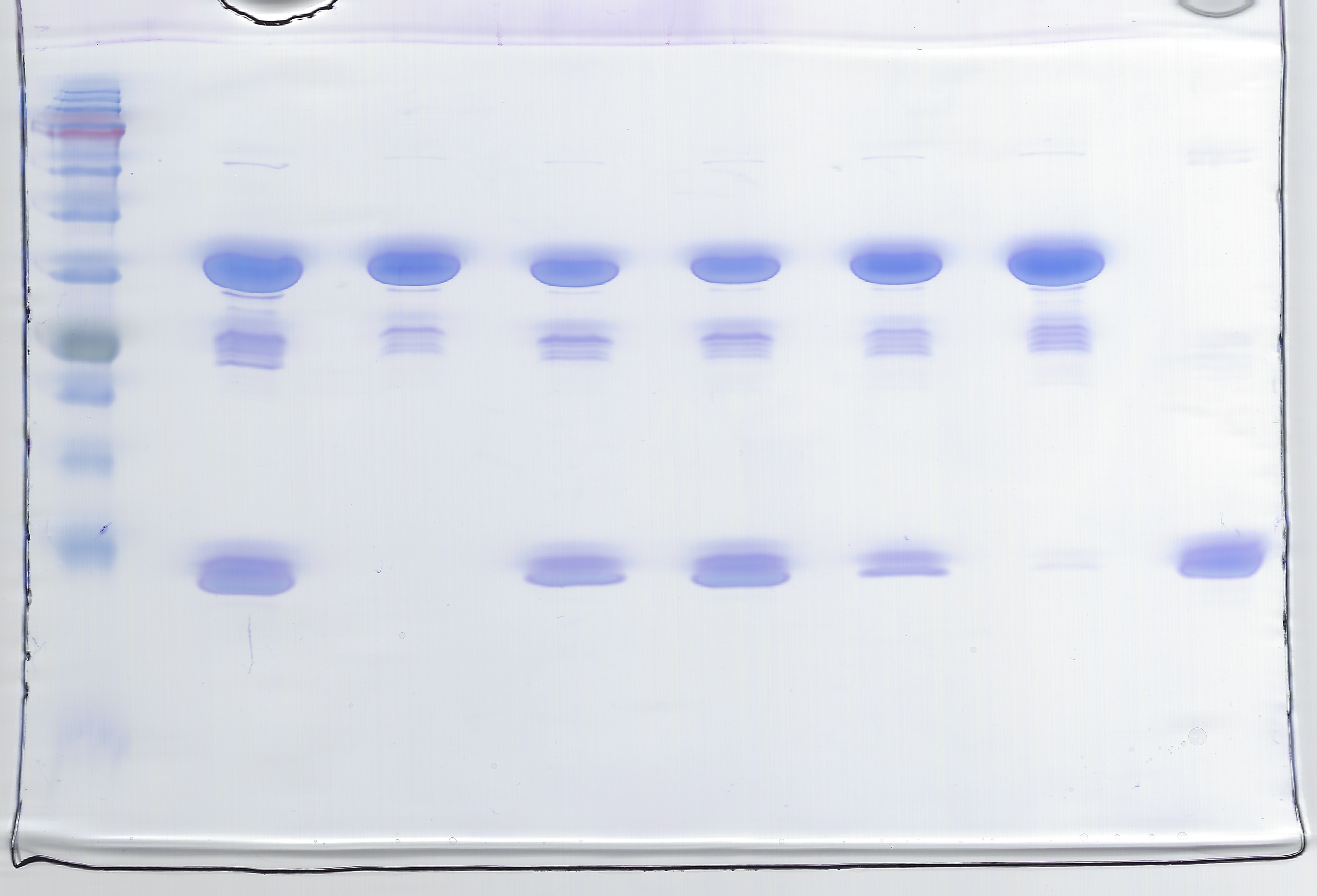

Supplement: Figure 7—source data 3. [file elife-74714-fig7-data3.zip › Figure 7-Source Data 3/Figure 7 - figure supplement 1 A/Figures for replicates/Figure 7 - figure supplement 1 A_rawdata2.3.png]

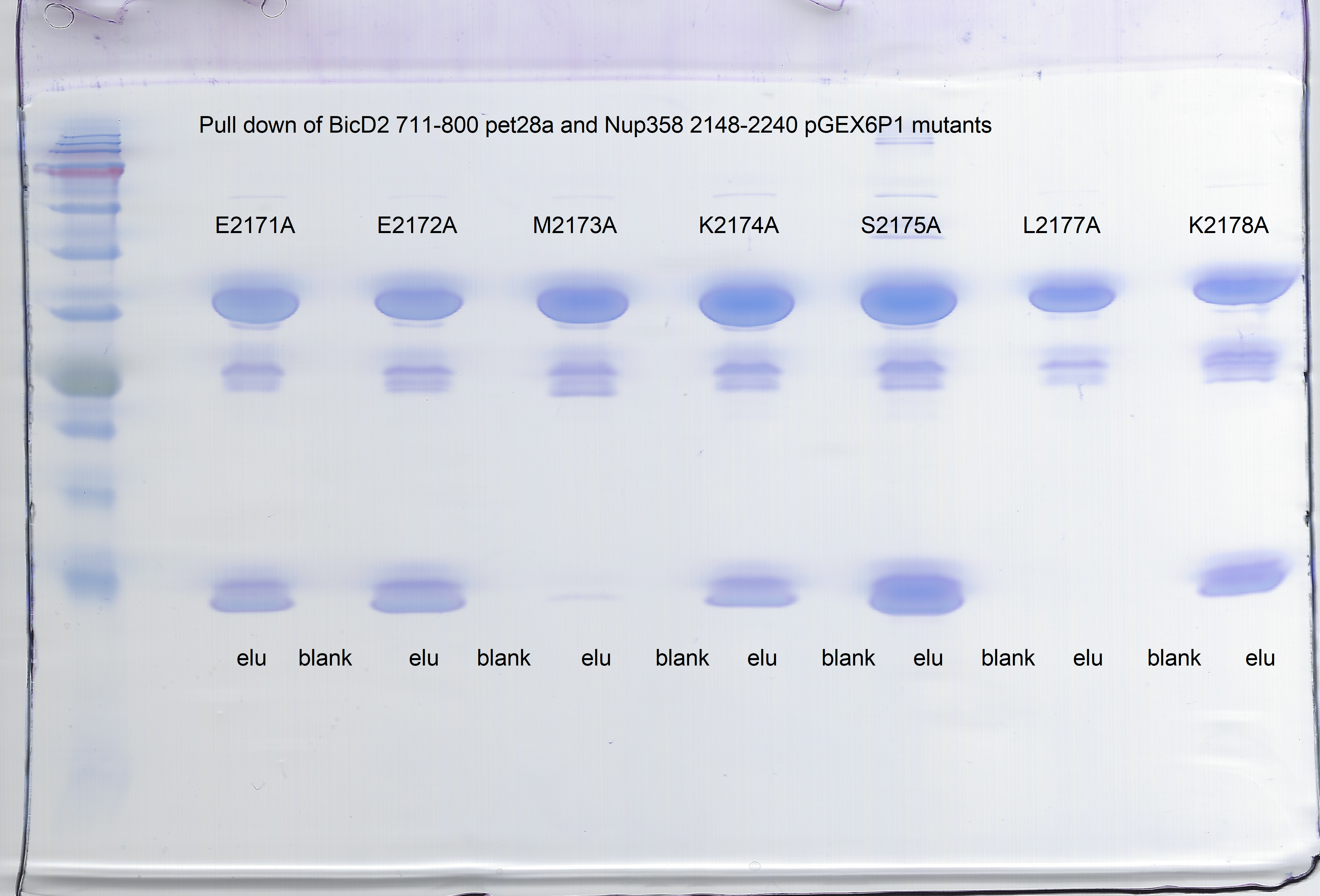

Supplement: Figure 7—source data 3. [file elife-74714-fig7-data3.zip › Figure 7-Source Data 3/Figure 7 - figure supplement 1 A/Figures for replicates/Figure 7 - figure supplement 1 A_rawdata2.2 labelled.png]

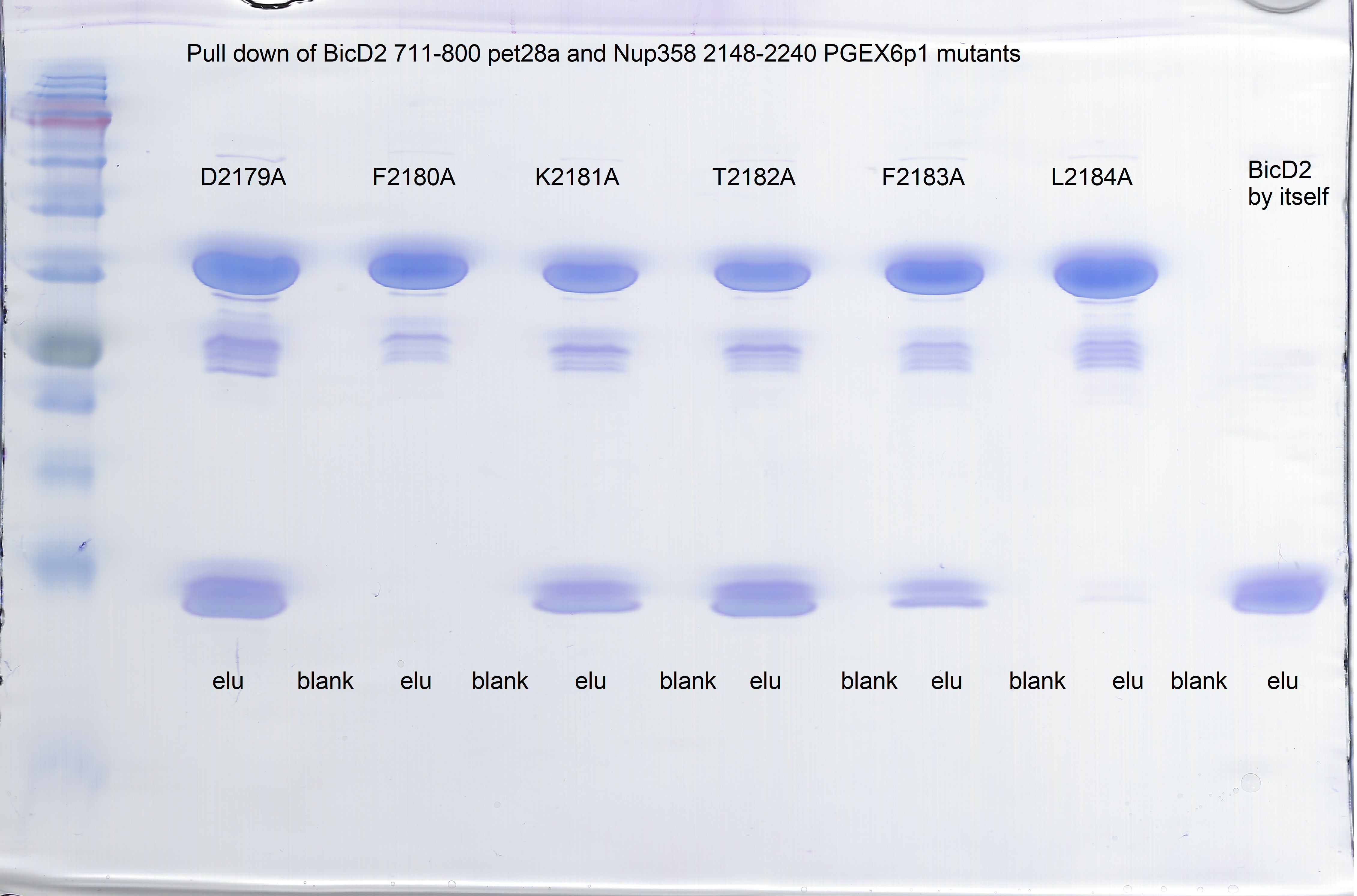

Supplement: Figure 7—source data 3. [file elife-74714-fig7-data3.zip › Figure 7-Source Data 3/Figure 7 - figure supplement 1 A/Figures for replicates/Figure 7 - figure supplement 1 A_rawdata2.3 labelled.png]

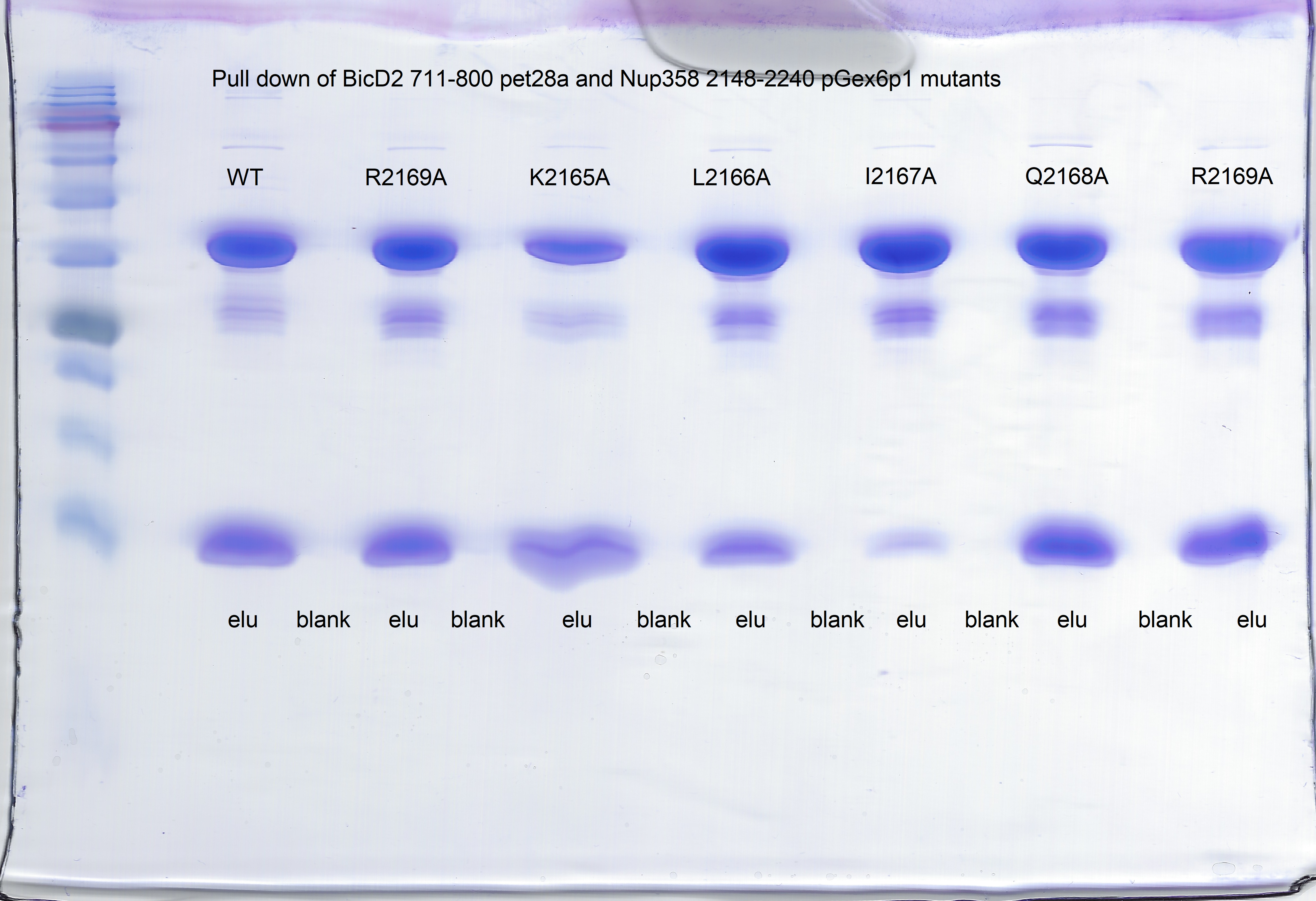

Supplement: Figure 7—source data 3. [file elife-74714-fig7-data3.zip › Figure 7-Source Data 3/Figure 7 - figure supplement 1 A/Figures for replicates/Figure 7 - figure supplement 1 A_rawdata2.1 labelled.png]

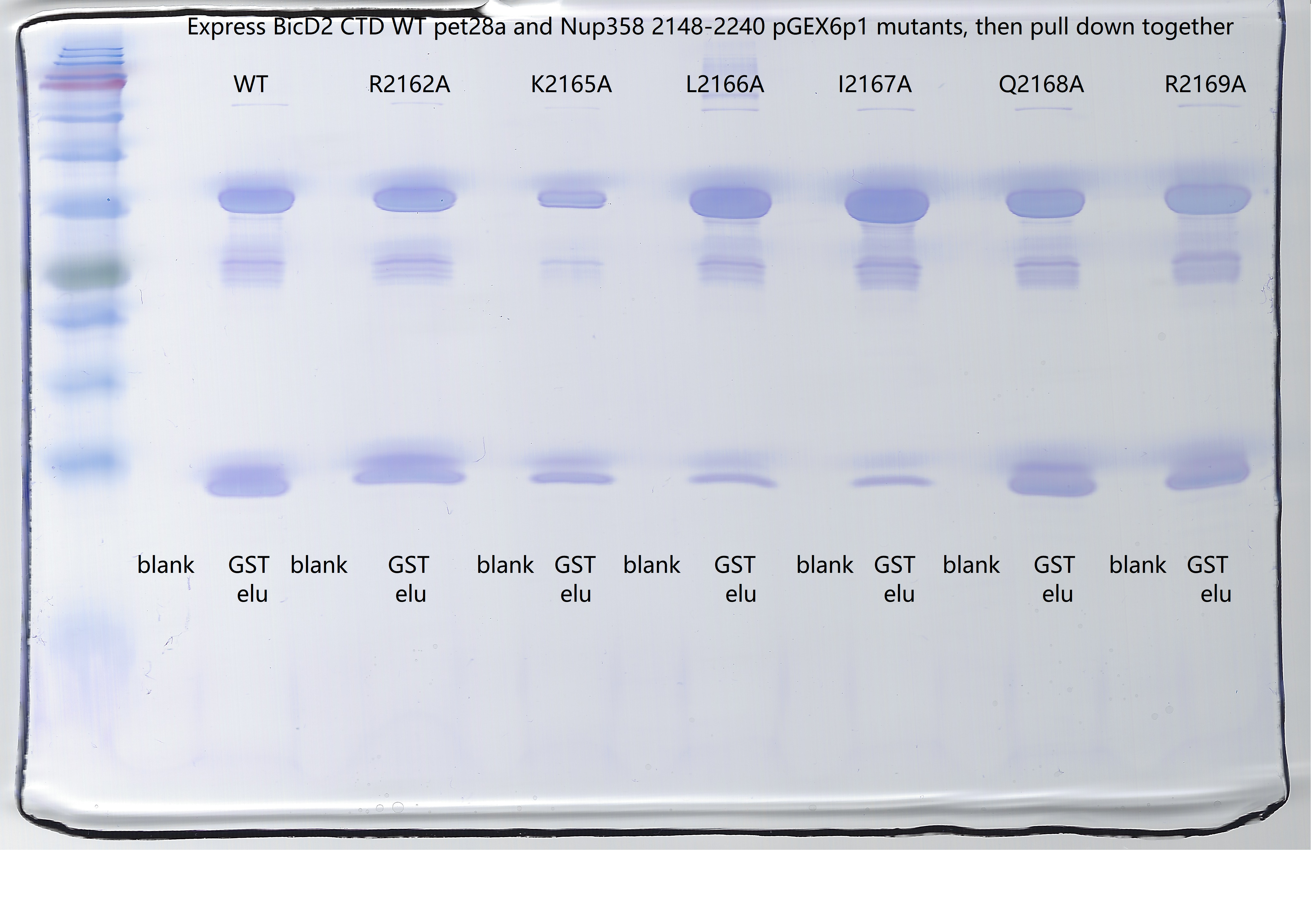

Supplement: Figure 7—source data 3. [file elife-74714-fig7-data3.zip › Figure 7-Source Data 3/Figure 7 - figure supplement 1 A/Figures for replicates/Figure 7 - figure supplement 1 A_rawdata1.1 labelled.png]

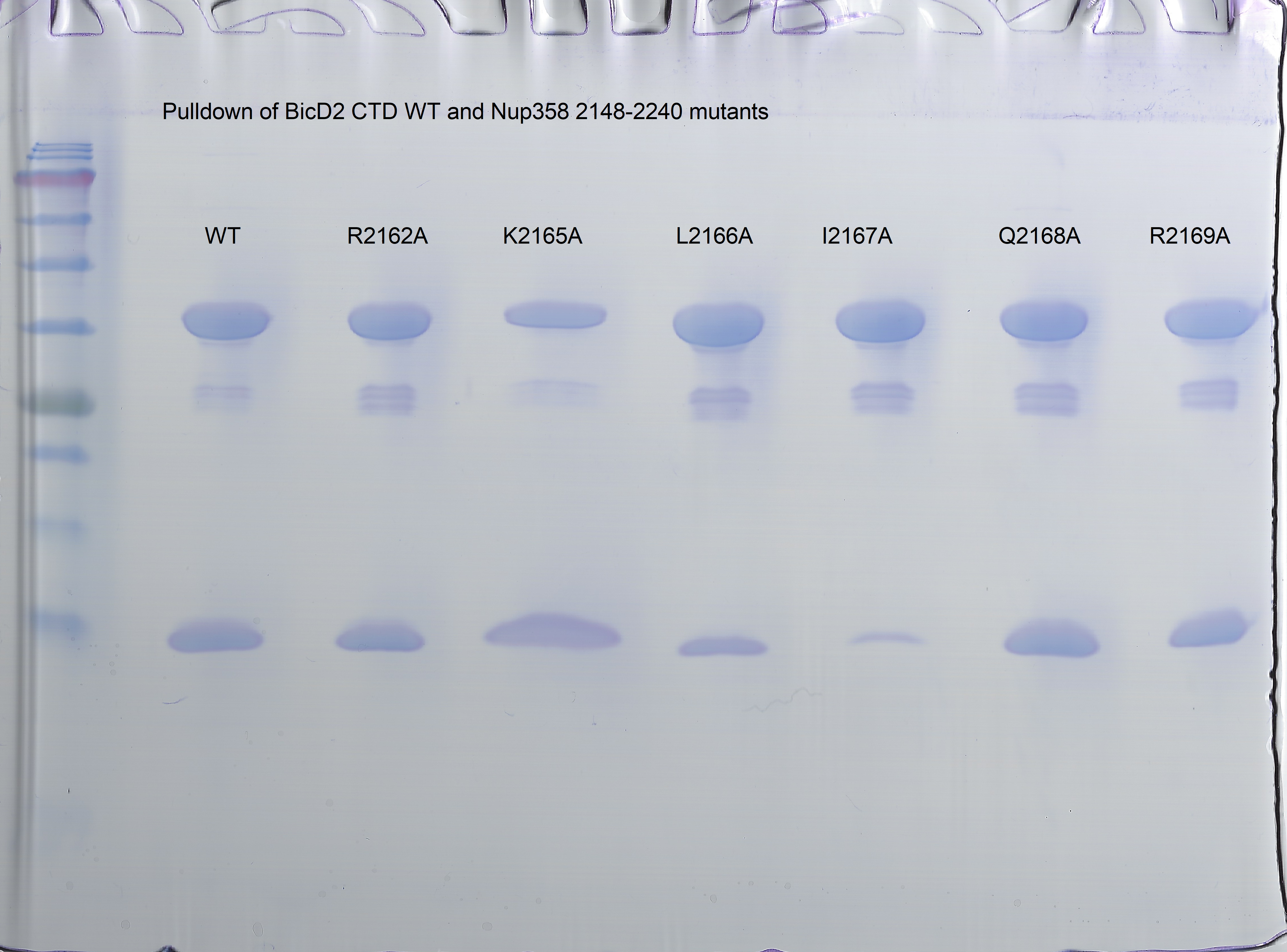

Supplement: Figure 7—source data 3. [file elife-74714-fig7-data3.zip › Figure 7-Source Data 3/Figure 7 - figure supplement 1 A/Figures used for the paper/Figure 7 - figure supplement 1 A_rawdata 1 label.png]

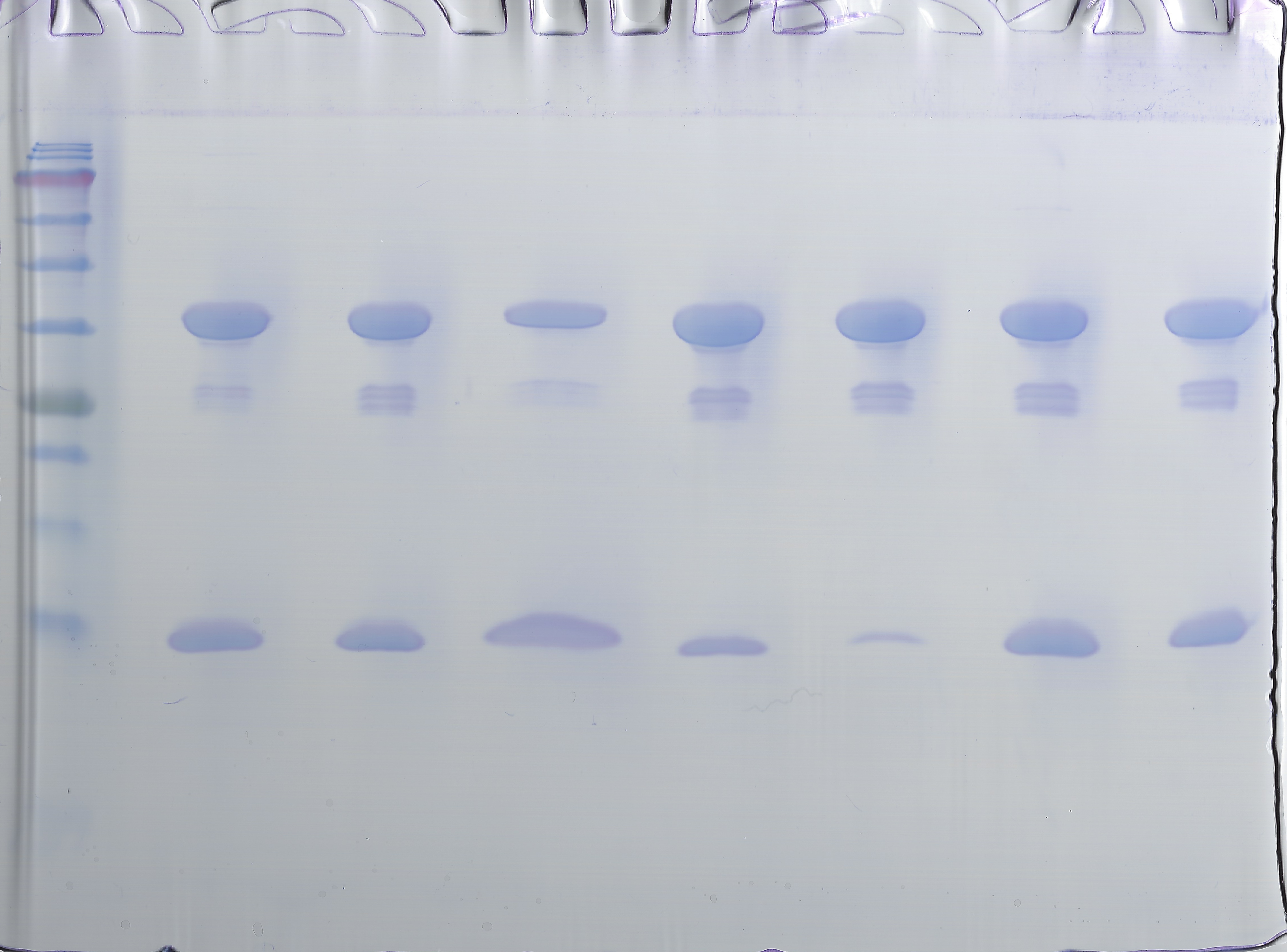

Supplement: Figure 7—source data 3. [file elife-74714-fig7-data3.zip › Figure 7-Source Data 3/Figure 7 - figure supplement 1 A/Figures used for the paper/Figure 7 - figure supplement 1 A_rawdata 1.png]

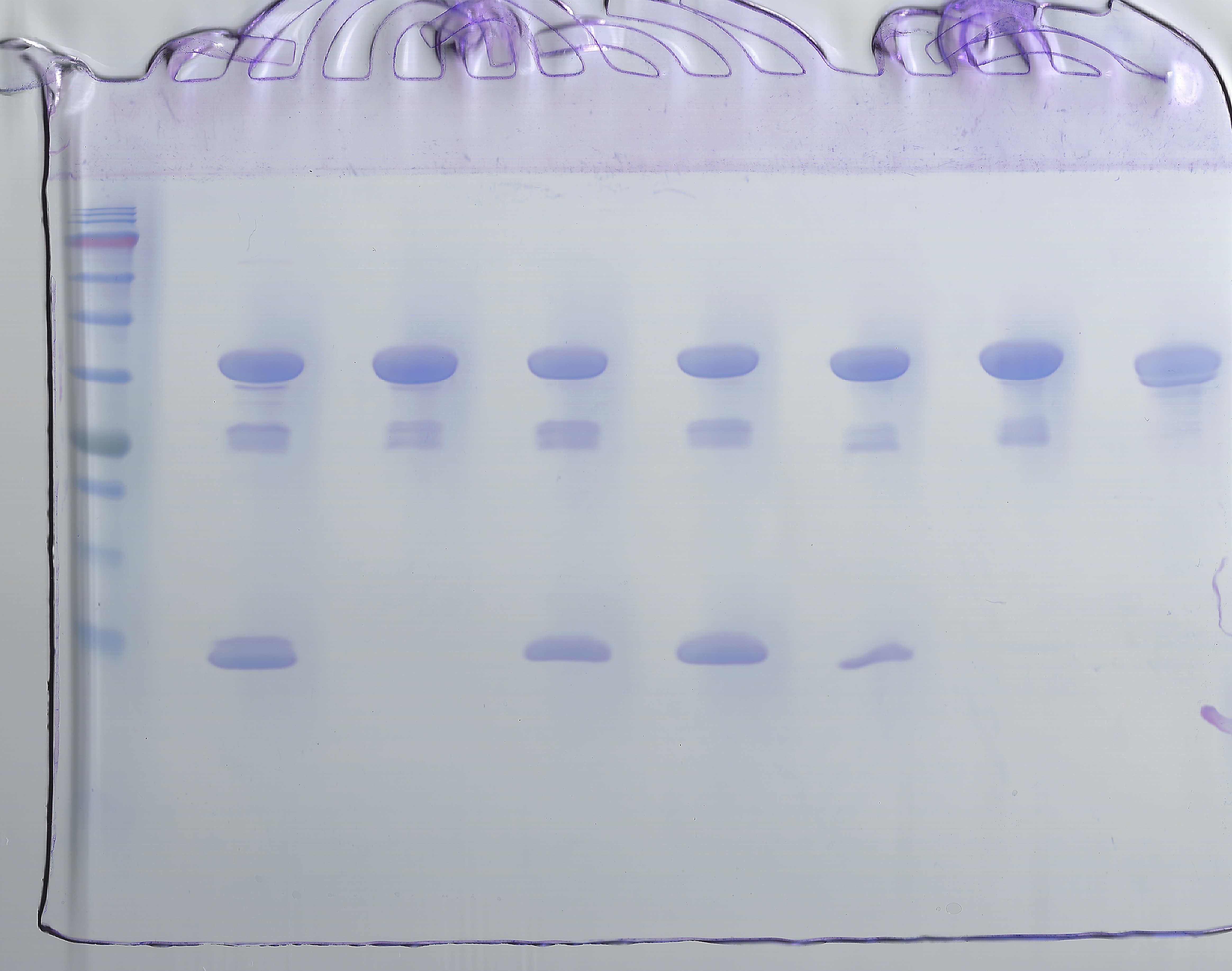

Supplement: Figure 7—source data 3. [file elife-74714-fig7-data3.zip › Figure 7-Source Data 3/Figure 7 - figure supplement 1 A/Figures used for the paper/Figure 7 - figure supplement 1 A_rawdata 3.png]

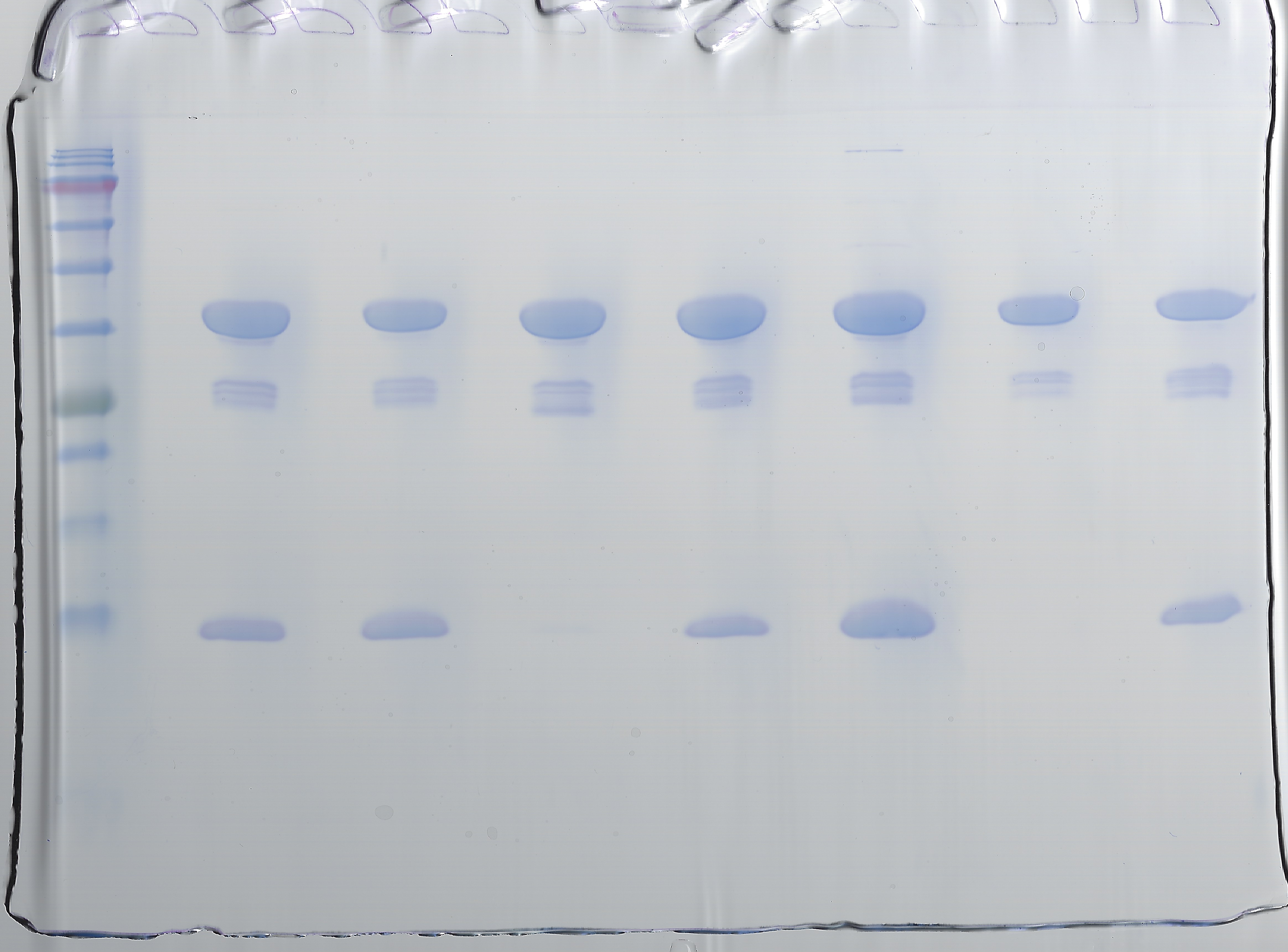

Supplement: Figure 7—source data 3. [file elife-74714-fig7-data3.zip › Figure 7-Source Data 3/Figure 7 - figure supplement 1 A/Figures used for the paper/Figure 7 - figure supplement 1 A_rawdata 2.png]

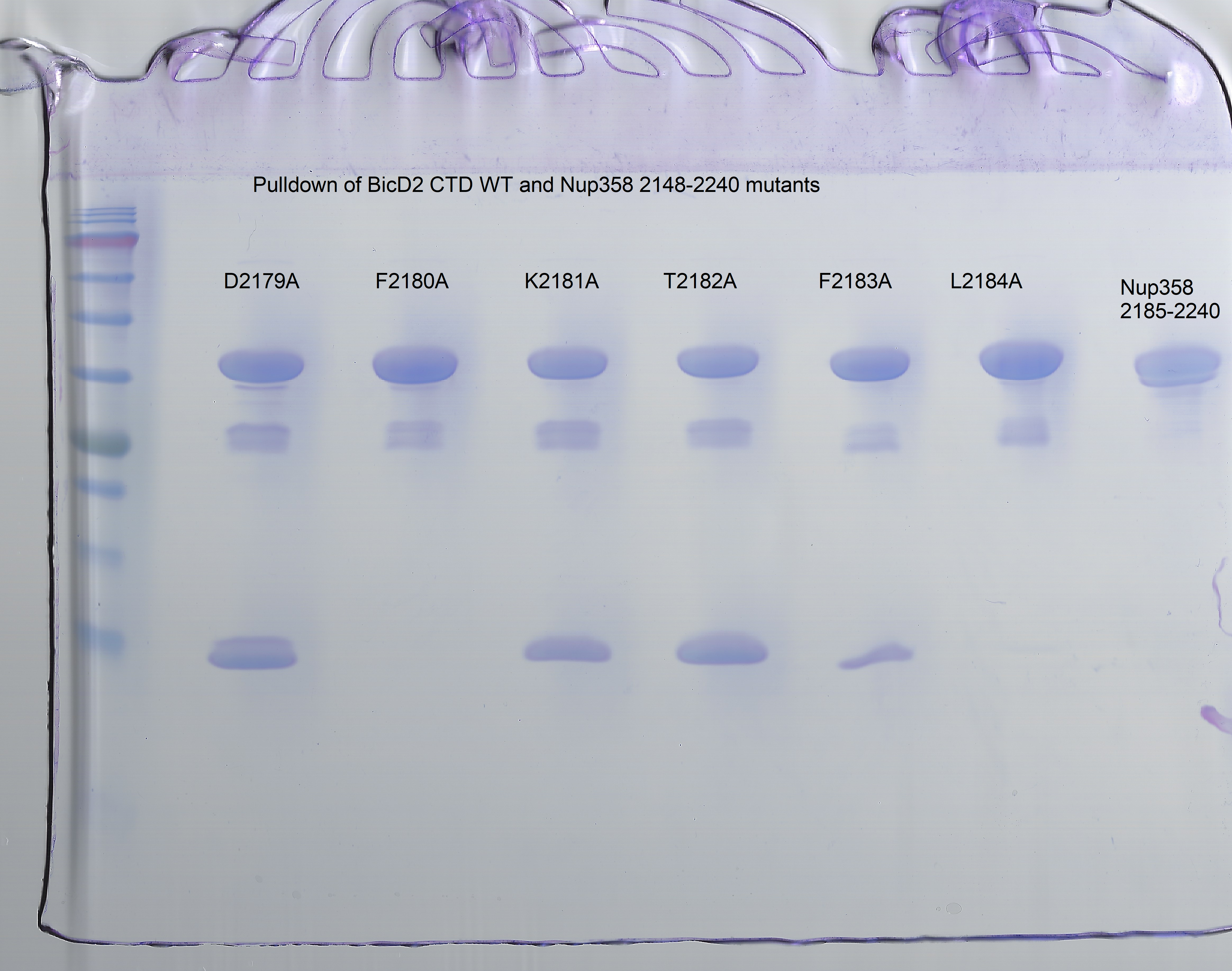

Supplement: Figure 7—source data 3. [file elife-74714-fig7-data3.zip › Figure 7-Source Data 3/Figure 7 - figure supplement 1 A/Figures used for the paper/Figure 7 - figure supplement 1 A_rawdata 3 label.png]

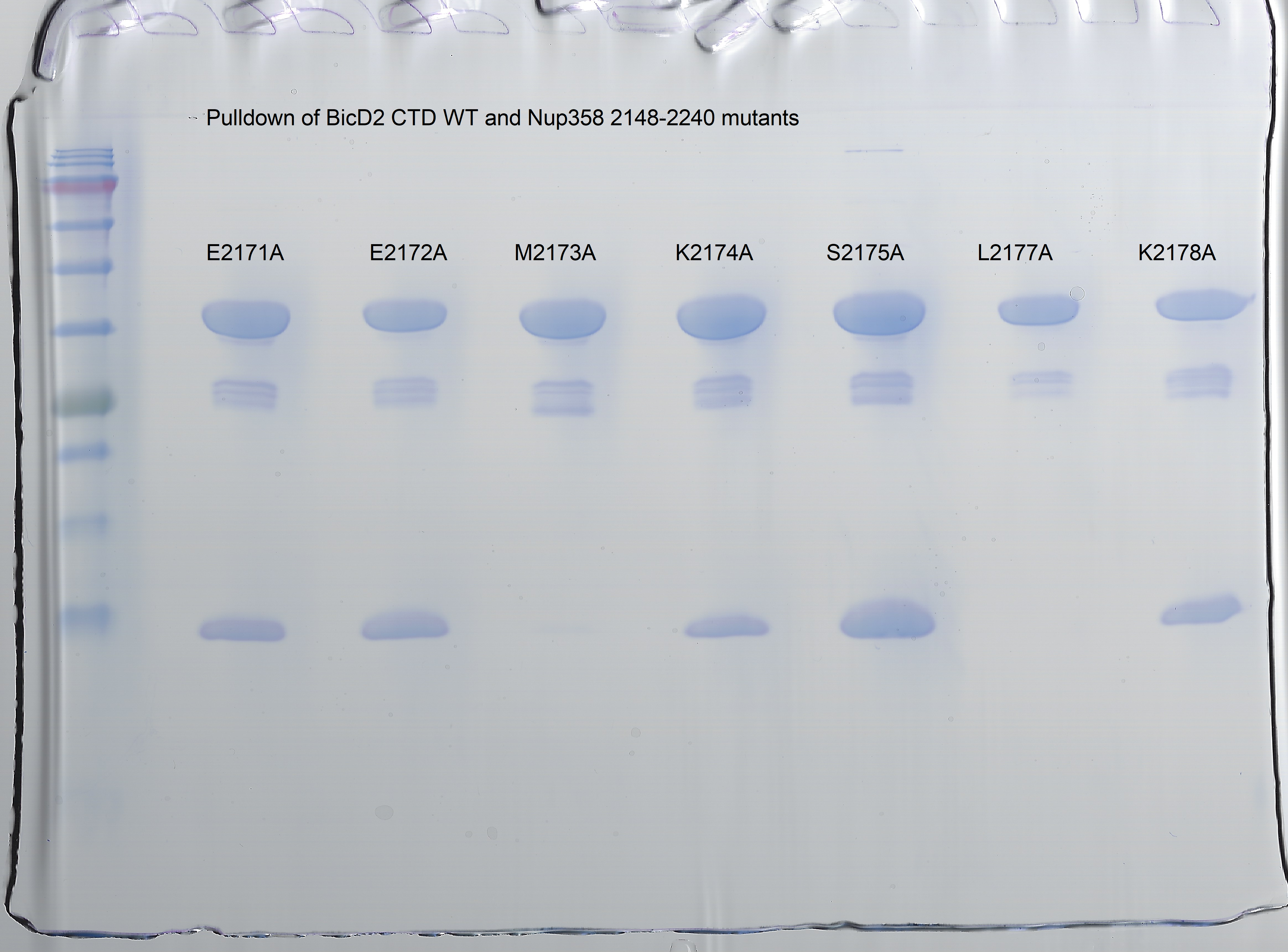

Supplement: Figure 7—source data 3. [file elife-74714-fig7-data3.zip › Figure 7-Source Data 3/Figure 7 - figure supplement 1 A/Figures used for the paper/Figure 7 - figure supplement 1 A_rawdata 2 label.png]
